# Bacteriophages in Pseudomonas aeruginosa evade the CRISPR-Cas I-F system by depletion of PAM sequences

**DOI:** 10.1099/mgen.0.001423

**Published:** 2025-06-17

**Authors:** Irene Ortega-Sanz, Alejandro Rubio, Antonio J. Pérez-Pulido

**Affiliations:** 1 Department of Food Technology, Safety and Health, Faculty of Bioscience Engineering, Ghent University, 9000 Gent, Belgium; 2 Department of Biotechnology and Food Science, University of Burgos, Burgos, Spain; 3 Andalusian Centre for Developmental Biology (CABD, UPO-CSIC-JA). Faculty of Experimental Sciences (Genetics Area), University Pablo de Olavide, 41013, Seville, Spain

**Keywords:** protospacers, protospacer adjacent motif, spacers, virus escape

## Abstract

Clustered regularly interspaced short palindromic repeats (CRISPR) and CRISPR-associated (Cas) proteins systems provide bacteria with an adaptive immune system against exogenous sequences, such as plasmids and bacteriophages (viruses of prokaryotes). To avoid autoimmunity, the recognition of a very short sequence called the protospacer adjacent motif (PAM) is essential for efficient immune response triggering. This raises the question of whether viruses targeted by certain CRISPR-Cas systems have a higher or lower frequency of their PAM sequences than non-targeted viruses. This was tested here in the opportunistic human pathogen *Pseudomonas aeruginosa* that presents five different types of CRISPR-Cas systems. The most frequent of them is the subtype I-F (present in 36% of the strains), which has the PAM 5′-CC sequence. When the viral genomes targeted by this system were analysed, their PAM frequency was found to be lower than that of non-targeted viruses. Remarkably, targeted viruses have comparatively lower G+C content. All this could be the result of selection pressure on the genome of these viruses to escape from the CRISPR-Cas I-F system.

**Impact Statement**

Fifty percent of sequenced *Pseudomonas aeruginosa* genomes possess clustered regularly interspaced short palindromic repeats and associated protein (CRISPR-Cas) adaptive immunity defence systems against bacteriophages (prokaryotic viruses or phages). However, a look at the spacers of these systems shows that not all phages are recognized by them. Here, we have found that at least in CRISPR-Cas subtype I-F systems, which are the most frequent in this bacterial species, the phages recognized by the immune system are subject to selection pressure in the protospacer adjacent motif sequence, whose consensus is the dinucleotide CC. In this way, recognized phages have a lower than expected CC frequency by chance, especially centred on the sequences of their protospacers (sequences recognized by the spacers).

## Data Availability

All data generated or analysed during this study are included in this published article. The code used to analyse the data and the supplementary files are available in the Zenodo repository: https://doi.org/10.5281/zenodo.15082351.

## Introduction

Prokaryotes have evolved multiple defence mechanisms against invading viruses (specifically called phages in bacteria) and plasmids, including clustered regularly interspaced short palindromic repeats and associated proteins (CRISPR-Cas), restriction modification and abortive infection [1]. Among these, CRISPR-Cas systems have become a revolutionary gene-editing technology thanks to essential discoveries that began in the last century [2,6]. These adaptive immunity systems consist of multiple spacers 23–50 bp long separated by repeats 17–84 bp long that comprise the CRISPR array encoding the RNA components, which is typically flanked by a locus of *cas* genes encoding proteins involved in the different phases at which these systems operate [7]. The length and sequence of the repeats and the length of the spacers show strong conservation within a CRISPR locus, but significant variations in the composition and number of spacers can be observed between arrays from the same organism or closely related strains [8]. Moreover, the selection pressure exerted by viruses on bacteria has led to the rapid evolution of CRISPR-Cas systems [9,10]. Therefore, a significant diversity of genes associated with CRISPR exists, which makes the classification of CRISPR-Cas systems challenging and even more so as sequencing data increase [11]. The current categorization of the CRISPR-Cas systems led to their classification into two classes (class 1 and class 2 systems) characterized by different effector module architectures [9]. These classes are further divided into three types each (class 1 includes types I, III and IV, and class 2 includes types II, V and VI) that are sub-grouped into 16 and 17 subtypes each, respectively, based on distinct Cas protein signatures (e.g. Cas3, Cas9 and Cas10 for types I, II and III, respectively). However, distinct CRISPR-Cas systems can coexist in the same organism, and in certain species, these loci can constitute a considerable part of the genome [8,11]. Class 1 CRISPR-Cas system is the most abundant in nature and comprises about 90% of CRISPR-Cas systems in bacteria and archaea [9,12]. Besides, type IV CRISPR-Cas systems are primarily found on plasmid-like elements and are specialized in targeting other plasmids [13]. It should be noted that CRISPR arrays are sometimes found in genomes lacking the corresponding *cas* genes, suggesting that they may have previously had a complete CRISPR-Cas system [14].

CRISPR-Cas systems confer highly adaptive and heritable immunity against exogenous DNA sequences in ~40% of bacteria and 90% of archaea, with a remarkable variability between phyla [9,15]. The CRISPR-Cas immune response can be divided into three phases: adaptation, expression and interference [8]. In the first stage, the system can integrate fragments of invading DNA sequences (called protospacers), such as phages, conjugative plasmids or mobile genetic elements, into CRISPR loci as spacers, serving as a molecular memory of past infections. During the expression stage, the CRISPR array is transcribed and processed into short mature CRISPR RNAs (crRNAs) containing a single spacer and parts of the flanking repeats. Finally, at the interference stage, the mature crRNA acts as a guide RNA that recognizes the protospacer of the corresponding invader, while directing the Cas endonuclease for protospacer cleavage and invader inactivation. Consequently, the CRISPR-Cas system provides specificity of the immune response to a particular foreign invader. However, spacer selection and acquisition are not random and require the recognition of a short DNA sequence motif (usually 2–6 bp in length) next to the protospacer known as the protospacer adjacent motif (PAM) by the adaptation module [16]. At the same time, the PAM sequence is also crucial for the recognition of the foreign sequence during the interference stage [17]. Even if the spacer is perfectly complementary to the target, the Cas proteins usually cannot cleave the target without a PAM. Thus, PAM recognition is of special significance for triggering the immune response after the phage or plasmid invades the bacterial cell, but also for preventing the CRISPR spacers from self-attacking [18].

CRISPR-Cas systems have been widely investigated in the group of bacteria known as ESKAPE, which are highly virulent and antibiotic resistant, including two Gram-positive bacteria (*Enterococcus faecium* and *Staphylococcus aureus*) and four Gram-negative bacteria (*Klebsiella pneumoniae*, *Acinetobacter baumannii*, *Pseudomonas aeruginosa* and *Enterobacter* spp*.*) [19]. Among these, extensive research has been performed in *Pseudomonas* species to understand the functionality of CRISPR-Cas systems [20,21]. Within this genus, *P. aeruginosa* is the most clinically important species and has become an emerging opportunistic human pathogen due to its ability to cause a wide variety of infections, particularly in vulnerable hospitalized patients [22]. All this evidence shows that *P. aeruginosa* has become an important model system for understanding the molecular mechanisms underlying CRISPR-Cas systems [23]. The genome of *P. aeruginosa* is large (> 6 Mbp), and ~50% of the sequenced *P. aeruginosa* genomes have been predicted to harbour a CRISPR-Cas system of subtypes I-C, I-E, I-F and IV, of which subtype I-F is by far the most frequent, appearing in 36% of the genomes [23,24]. Furthermore, several approaches have been applied to predict the PAM recognized by distinct *P. aeruginosa* CRISPR-Cas systems, although with a limited set of strains from 1 to 44 [20,25, 26]. This requirement of the PAM sequence gives rise to the idea that viruses and plasmids recognized by a given CRISPR-Cas system may have a different nucleotide composition compared to non-recognized sequences. In particular, it has already been proven that phages recognized by CRISPR-Cas systems could escape from CRISPR-Cas systems by the mutational pressure on their PAM sequences [27,29]. To test this hypothesis, a large *P. aeruginosa* pangenome comprising almost 8,000 genomes of this species was analysed for the presence of PAMs in plasmids and viruses recognized by the different CRISPR-Cas systems and compared to that of any other foreign sequences not recognized by this immune system. Thus, it was found that viral genomes recognized by the CRISPR-Cas I-F system present a lower frequency of PAM 5′-CC sequences, as well as a reduced G+C content, suggesting positive evolution to escape from this defence system.

## Methods

### Spacers collection from CRISPR arrays

A total of 7,876 *P*. *aeruginosa* genomes were retrieved from the National Center for Biotechnology Information (NCBI) Genome database on 14 June 2021 in a previous work developed by Rubio *et al*. [19]. In brief, CRISPRCasFinder v4.2.20 was used with default configuration to identify the spacers of the *P. aeruginosa* CRISPR-Cas systems [30], while CRISPRCas-Typer v1.4.1 was used to determine the subtype of the CRISPR-Cas system [31]. A genome was annotated with a given CRISPR-Cas system when all the *cas* genes of the adaptation and interference modules were present. In the case of type IV systems, only the interference module was verified since they typically lack Cas1-Cas2 adaptation modules. To obtain the CRISPR arrays, the JSON file from CRISPRCasFinder and the crisprs_all.tab file from CRISPRCasTyper were subsequently used. Only those spacers with known subtype and array orientation, as well as evidence level equal to 4 by CRISPRCasFinder, were considered for further analysis. In addition, a maximum 100 bp window size was considered as CRISPR array location mismatches between CRISPRCasFinder and CRISPRCasTyper for CRISPR-Cas subtype assignment, as well as mismatches in the length of repeat sequences.

### PAM prediction

For each CRISPR-Cas system subtype, the PAM was predicted through the Spacer2PAM v0.0.0.9000 package of R v4.2.2 (https://www.R-project.org/) using the ‘Comprehensive’ method with default parameters [32]. Briefly, the CRISPR-Cas system’s host organism name and subtype were passed to setCRISPRinfo to assign the name of the CRISPR-Cas system and the output file name. Then, a FASTA file with the spacer sequences and appropriate headers was generated with df2fasta using a formatted dataframe with the spacer data containing the headers ‘Strain’, ‘Spacers’, ‘Array.Orientation’, ‘Repeat’, ‘Array’ and ‘Spacer’. Next, the spacer sequences were aligned using the blast web interface with the blastn algorithm and excluding eukaryotes (taxid: 2759). The resulting hit table was downloaded in .csv format and was passed to alignmentCSV2DF to convert it into a dataframe. The resulting dataframe was then passed to joinSpacerDF and AlignmentDF to join the spacer data to each alignment in the hit table while assigning the accession number of the genus and species name of the organism that encodes each alignment sequence using the taxonomizr v0.9.3 package (https://cran.r-project.org/package=taxonomizr). The resulting joined dataframe was finally passed to join2PAM for PAM prediction over the range of 256 filter criteria used by Rybnicky *et al*. [32]. These filters removed alignments based on the number of gaps present in the alignment (> 0, 1, 2 or 3), *E* value of the alignment (> 0.01, 0.05, 0.5 or 1), the length of the alignment compared to the length of the spacer (> 0, 1, 3 or 5 nucleotide of difference) and the start of the query sequence relative to the spacer sequence start (> 1, 2, 5 or 7 nucleotide positions). PAM sequences with a prediction score above a 75th percentile threshold were selected as functional PAMs.

### Evaluation of PAM sequences in plasmids and phages

The PLSDB database v2020_06_23_v2 and the IMG/VR v3 high-quality genome database (IMG_VR_2020-10-12_5.1) [33,34], with sequences of bacterial plasmids and viral genomes, respectively, were employed to identify and verify the consensus PAM in plasmids and viruses. Redundancy was eliminated by sequence-level clustering using the CD-HIT-EST v4.8.1 algorithm with a 95% identity threshold. Plasmids or viruses with a sequence identity ≥95% (or 100% in a later analysis) and a query coverage=100% against a spacer of a *P. aeruginosa* CRISPR-Cas system subtype were identified as being targeted by such CRISPR-Cas system subtype, while non-targeted in other cases. In the targeted sequences, the flanking sequence upstream of the protospacer (10 nt) was extracted to create the DNA logo with the ggseqlogo v0.1 package of R v4.2.2 [35]. The occurrence of PAM sequences differing at the significant positions previously determined by Spacer2PAM was calculated in the plasmids and viruses being targeted by the *P. aeruginosa* CRISPR-Cas systems.

### Determination of the frequency of PAM sequences in plasmids and viruses

The occurrence of the consensus PAM predicted by Spacer2PAM in the forward and reverse complement directions (number of PAM sequences in both directions/number of *k*-mers of same length as PAM sequence in both directions) and the G+C content were calculated in each group of targeted/non-targeted plasmids or viruses, considering that these molecules were double-stranded. The Shapiro–Wilk test was performed to check for normality. The non-parametric Wilcoxon rank-sum test (also called the Mann–Whitney *U* test) with significance *P* < 0.05 was used to determine whether there was any statistically significant difference in the frequency of PAMs or G+C content between the groups of plasmids or viruses targeted/non-targeted by each CRISPR-Cas system. The Holm method was used to adjust the *P*-value for multiple testing.

## Results

### Search for PAM sequences

**Fig. 1.**
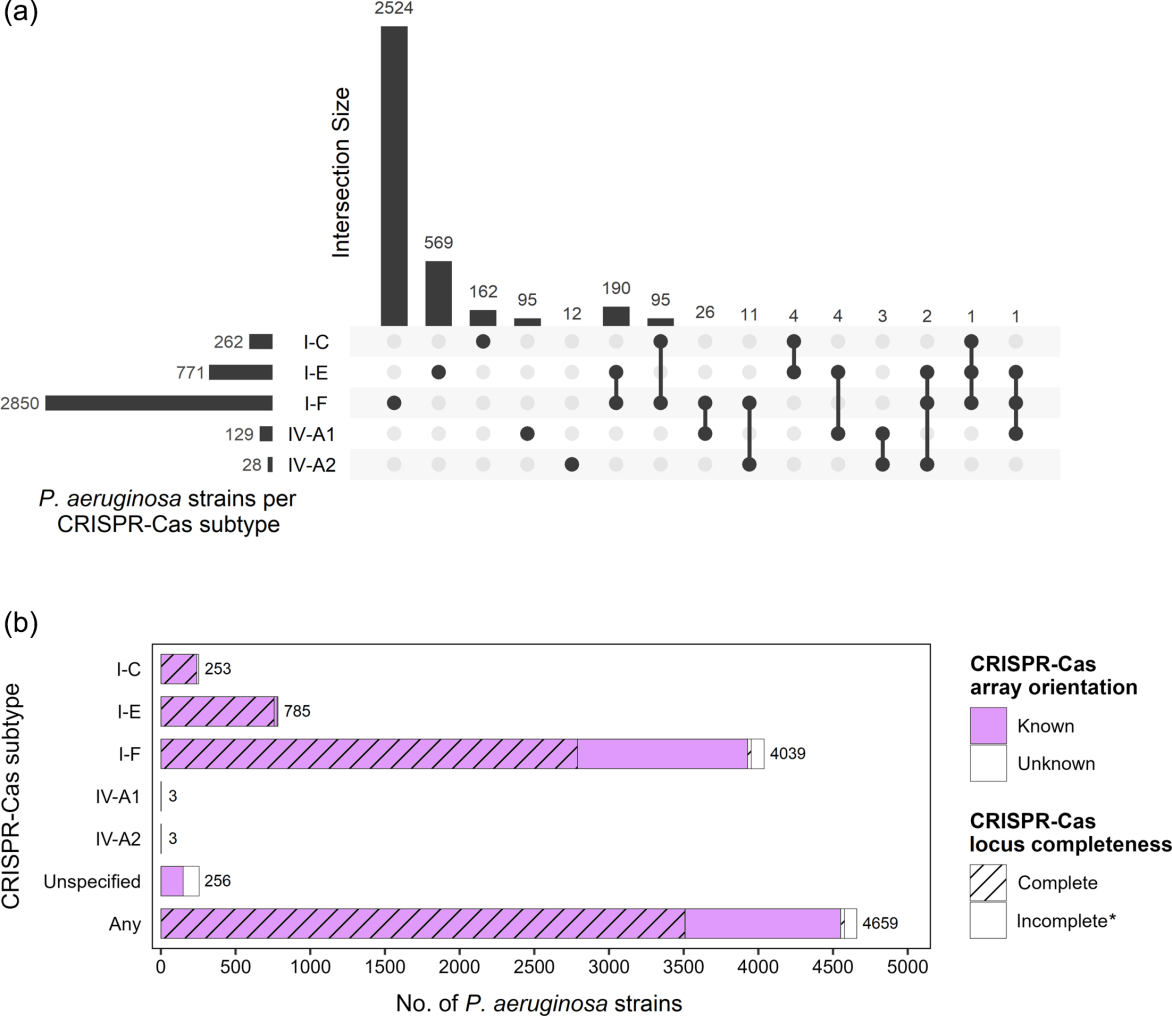
Distribution of CRISPR-Cas systems of *P. aeruginosa* grouped by subtype. (**a**) Candidate CRISPR-Cas systems and co-occurrence between CRISPR-Cas subtypes in the same genome. (**b**) Proportion of *P. aeruginosa* strains harbouring CRISPR arrays. *Most incomplete CRISPR-Cas systems present only CRISPR arrays, and others miss one or more *cas* genes.

**Fig. 2.**
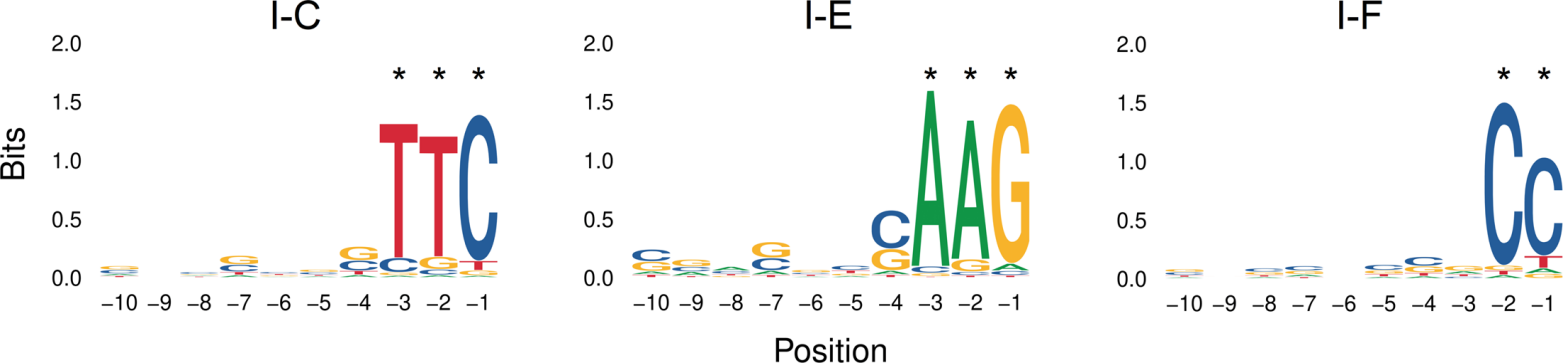
Representative sequence logo of the upstream PAM sequences for each CRISPR-Cas system according to Spacer2PAM. Significant positions are marked with an asterisk.

To begin with, a total of 7,876 *P*. *aeruginosa* genomes were analysed, of which 3699 had putative CRISPR-Cas systems of the types I and/or IV. Of those, subtype I-F appeared in 36% of genomes, followed in frequency by subtypes I-E and I-C (10% and 3%, respectively), while subtypes IV-A1 and IV-A2 were present in <2% of all genomes (Fig. 1a). Moreover, co-occurrence of two or three CRISPR-Cas systems was found, especially involving the most frequent subtype I-F. A total of 190 genomes had both subtypes I-F and I-E, and 95 genomes had both subtypes I-F and I-C. Furthermore, 1,248 genomes had isolated CRISPR arrays without the associated *cas* genes next to them, and 256 other genomes harboured unspecified CRISPR arrays (Fig. 1b). To establish the PAM sequences of the different types of *P. aeruginosa* CRISPR-Cas systems, all the CRISPR arrays found in the *P. aeruginosa* genomes were initially taken to have a set as complete as possible. However, CRISPR arrays of poor quality or in which neither the orientation nor the subtype could be determined were filtered out (see ‘Methods’). Thus, a total of 9,857 CRISPR arrays were finally considered (Table 1), appearing in a total of 4,526 *P*. *aeruginosa* strains (57%). The number of CRISPR arrays was almost twice the number of strains presenting arrays, as the CRISPR-Cas type I-F usually presented two arrays appearing on either side of the *cas* gene cluster. In particular, CRISPR arrays of subtype I-F were predominant and present in more than 50% of the 7,876 *P*. *aeruginosa* genomes, while subtypes IV-A1 and IV-A2 were rare (<1%), with only six genomes presenting CRISPR arrays. Therefore, the subtypes IV-A1 and IV-A2 were discarded from subsequent analysis for being underrepresented. The resulting 9850 arrays representing type I CRISPR-Cas system comprised 151,819 spacers from the initial number of 161,377 (94%) (Table S1, available in the online Supplementary Material) that were further used to predict the PAM sequence identified by each CRISPR-Cas system (Table 1), which resulted to be invariable within each of them (Table S2). Finally, the PAM sequences found upstream of the protospacers were used to construct the sequence consensus, proposing the following sequences: 5′-TTC (subtype I-C), 5′-AGG (subtype I-E) and 5′-CC (subtype I-F) (Fig. 2).

**Table 1.** Summarized upstream PAM prediction for each CRISPR-Cas system found in *P. aeruginosa* strains from the NCBI Genome database

CRISPR subtype	Array used (%)	Array direction	No. of spacer (%)	No. of protospacer (%)	Comprehensive PAM prediction	Consensus PAM prediction
I-C	242 (2.46)	122 F120 R	5,703 (3.75)	323,659 (2.68)	5′-NNNNNNNTTC	5′-TTC
I-E	1,492 (15.14)	760 F732 R	20,059 (13.20)	1,259,635 (10.43)	5′-NNNNNNNAAG	5′-AAG
I-F	8,116 (82.34)	4,051 F4,065 R	126,057 (82.98)	10,494,594 (86.87)	5′-NNNNNNNNCC	5′-CC
IV-A1	4 (0.04)	2 F2 R	34 (0.02)	415 (< 0.01)	5′-NT/CNNNNNA/CNN5′-NNNNNNNA/CA/GN*5′-A/TNNNNNNA/CA/GN5′-NT/CNNNNNA/CA/GN*	5′-MNN
IV-A2	3 (0.03)	1 F2 R	57 (0.04)	2,141 (0.02)	5′-NNANNNNNNC*5′-NGANNNNNNC*	5′-ANNNNNNC
Total	9,857 (100)	4,933 F4,917 R	151,910 (100)	12,080,444 (100)	–	–

F, forward. R, reverse. Sequences in italics were below the 75th percentile PAM score threshold and were discarded for the consensus PAM prediction.

### Evaluation of the consensus PAM sequence in plasmids and phages

**Fig. 3.**
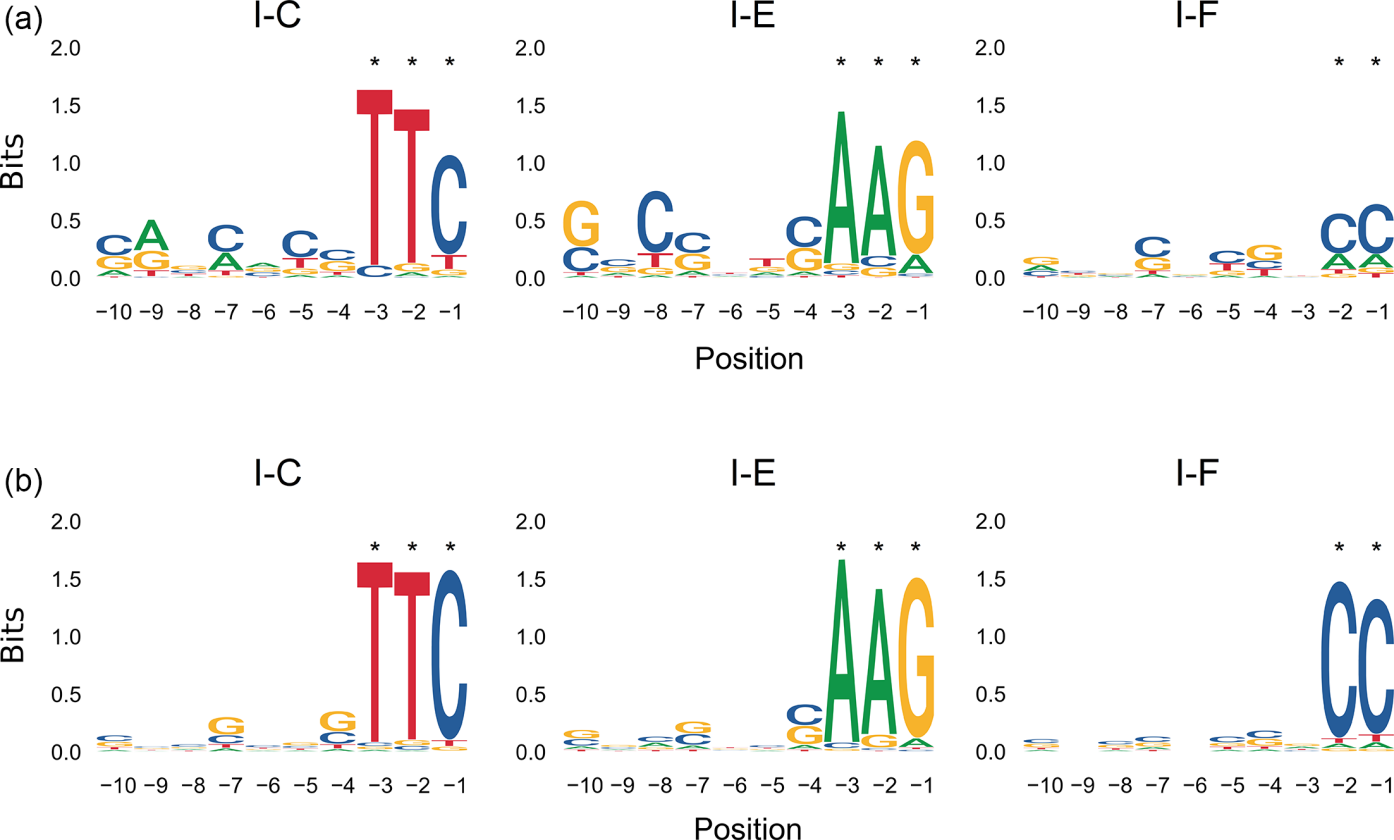
Representative DNA logos for the upstream PAM sequence relative to the protospacers in (**a**) the plasmids and (**b**) the viruses targeted by each *P. aeruginosa* CRISPR-Cas system subtypes I-C, I-E and I-F. Significant positions are marked with an asterisk.

The consensus PAM sequences previously predicted were verified in two different cohorts of sequences infecting *P. aeruginosa*. These were the PLSDB database for plasmid sequences and the IMG/VR database for viral genomes. A total of 150 non-redundant sequences out of 157 plasmids identified in *P. aeruginosa*, and 1,487 non-redundant sequences out of 3,469 viruses identified in *P. aeruginosa* were selected. The spacers within the CRISPR arrays representing each *P. aeruginosa* type I CRISPR-Cas system (I-C, I-E and I-F) were used as recognition elements to find matching plasmid and viral genomes, which were classified as being targeted foreign invaders by these immune systems or non-targeted sequences, using an identity threshold of 95%. A total of 87 plasmids (58%) were targeted by any of the *P. aeruginosa* type I CRISPR-Cas systems (I-C, I-E and/or I-F), 23 of which (26%) were targeted by all three of them (Fig. S1A). In contrast, a higher proportion of viruses was targeted by the three subtypes (73%) (Fig. S1B), while only 60 sequences out of 1,487 viruses were not recognized by any of them (4%). In both groups, most plasmids and phages being targeted by these CRISPR-Cas systems were identified by CRISPR-Cas subtype I-F (84 plasmids and 1,420 viruses), while *P. aeruginosa* CRISPR-Cas system I-C targeted the lowest number of foreign invaders (25 plasmids and 1,085 viruses). For each *P. aeruginosa* type I CRISPR-Cas system, the PAM sequences in the plasmids and phages being targeted by each of them were 5′-TTC for subtype I-C, 5′-AAG for subtype I-E and 5′-CC for subtype I-F (Fig. 3). However, the information content of these positions (measured in bits) was higher in phages.

### The sequences targeted by the I-F subtype showed the most diversity of PAMs

The genomes of the plasmids and viruses that were previously identified as being targeted by the *P. aeruginosa* CRISPR-Cas system were subjected to further analysis. Among these, higher occurrences of the consensus PAM sequences predicted by Spacer2PAM (Table 1) were found in viral genomes ranging from 83 to 87%, despite showing a higher diversity of PAMs (Supplementary Fig. S2). In contrast, the occurrence was lower in the case of the plasmids (varying from 50 to 75%), especially for subtype I-F, which had only 50% of the PAM sequences matching the consensus PAM previously predicted (5′-CC). On the other hand, the PAM sequence 5′-TTC recognized by subtype I-C was found with the highest proportions in both plasmids (75%) and viruses (87%). The highest diversity of PAM sequences targeted by the *P. aeruginosa* CRISPR-Cas systems was found for subtype I-F, where all the possible 16 combinations of PAMs were detected in plasmids and viruses, opposite to subtypes I-E and I-C. For subtype I-E, 36 and 58 different PAMs out of 64 possible trinucleotide combinations were found in the recognized plasmids and viruses, respectively, while 15 and 51 different PAMs were found in the recognized plasmids and viruses targeted by subtype I-C, respectively (Table S3, Fig. S3). Moreover, the PAM sequences that maintained the same proportion of G+C content in the viruses recognized by subtype I-F (5′-GG, 5′-CG or 5′-GC) accounted for only 2.8% of all PAM sequences found (0.1%, 1.1% and 1.6%, respectively) (Table S3, Fig. S3).

### Consensus PAM sequences appear less frequently in the sequences targeted by the I-F subtype

**Fig. 4.**
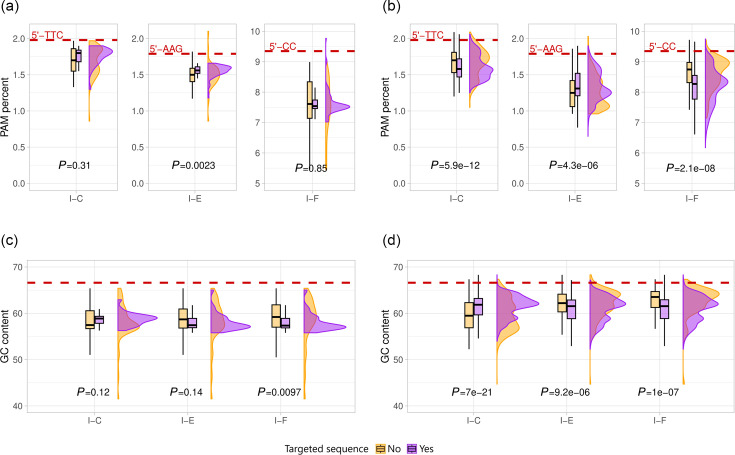
Distribution of PAM frequencies and G+C content in the groups of targeted and non-targeted sequences by the *P. aeruginosa* CRISPR-Cas subtypes I-C, I-E and I-F: (**a**) PAM frequency for plasmids, (**b**) PAM frequency for phages, (**c**) G+C content for plasmids and (**d**) G+C content for phages. Dashes lines show the PAM frequency or G+C content in the *P. aeruginosa* reference strain PAO1 (GCF_000006765.1). The Holm method was used to adjust the *P*-value for multiple comparisons. Targeted sequences are shown in purple and non-targeted sequences are shown in orange. The numerical values can be found in Table S4.

Variable frequencies of the consensus PAM sequences were observed between the recognized and non-recognized plasmids and viruses by the *P. aeruginosa* type I CRISPR-Cas systems (I-C, I-E and I-F) (Fig. 4). Overall, higher PAM frequencies were observed in the plasmids and viruses targeted by *P. aeruginosa* CRISPR-Cas subtype I-F, followed by CRISPR-Cas subtype I-C, and lower frequencies were found in the sequences targeted by CRISPR-Cas subtype I-E (Table 2). However, it should be reminded that the probability of occurrence of a motif 2-nt long (PAM sequence of subtype I-F) is higher (1/16) than that of motifs 3-nt long (PAM sequences of subtypes I-E and I-C) (1/64). Moreover, the frequencies of the consensus PAM sequences between the plasmids targeted by the *P. aeruginosa* CRISPR-Cas systems and those not targeted were similar, while larger differences were observed for viral sequences, especially for the subtypes I-C and I-F, which showed the most significant variations in the PAM frequency with *P*-values of 5.9e−12 and 2.1e−8, respectively. In particular, for these two subtypes, a reduction in the occurrence of the PAM sequence in the recognized viruses was detected, opposite to subtype I-E. Moreover, such a reduction was higher in the case of the abundant subtype I-F, for which the median PAM frequency in the targeted phages by this system differed by 0.47% compared to those not targeted (8.27% vs. 8.74%) (Table 2). Furthermore, 22% of the non-targeted viral genomes by the subtype I-F showed PAM occurrences higher than 9%, which were close to the frequency of this dinucleotide (5′-CC) in the reference *P. aeruginosa* PAO1 genome (GCF_000006765.1) (9.35%).

**Table 2.** Summarized information of the plasmids and viruses targeted by each subtype of *P. aeruginosa* CRISPR-Cas system Targeted plasmids and viruses were defined as those with a sequence identity ≥95% and a query coverage=100% against a spacer of a *P. aeruginosa* CRISPR-Cas system, while non-targeted in other cases.

		Recognized plasmid/virus				Non-recognized plasmid/virus		
Database	CRISPR-Cas subtype	Number of protospacers	Number of different sequences	Frequency of consensus PAM (%)*^†^	G+C content (mol%)*†	Number of different sequences	Frequency of consensus PAM (%)*†	G+C content (mol%)†
PLSDB (*n*=150)	I-C	2,579	25	1.80 (1.68 to 1.84)	58.87 (57.84 to 59.35)	125	1.73 (1.55 to 1.87)	57.44 (56.63 to 60.53)
	I-E	5,813	65	1.56 (1.52 to 1.61)*	57.43 (56.89 to 58.90)	85	1.50 (1.42 to 1.59)	58.68 (56.70 to 60.86)
	I-F	85,793	84	7.54 (7.46 to 7.74)	57.35 (56.85 to 58.90)	66	7.59 (7.07 to 8.34)	58.93 (56.76 to 61.57)‡
IMG/VR (*n*=1,487)	I-C	77,274	1,085	1.58 (1.47 to 1.72)	61.85 (59.66 to 63.20)¶	402	1.70 (1.53 to 1.81)§	59.48 (56.87 to 62.30)
	I-E	318,491	1,345	1.31 (1.21 to 1.52)**	61.54 (58.84 to 62.87)	142	1.25 (1.06 to 1.42)	62.20 (60.31 to 64.13)§
	I-F	2,260,931	1,420	8.27 (7.77 to 8.55)	61.54 (58.87 to 62.92)	67	8.74 (8.27 to 8.98)**	63.52 (61.26 to 64.70)**

*The following consensus PAMs were searched: 5′-TTC for subtype I-C, 5′-AAG for subtype I-E and 5′-CC for subtype I-F.

†Results are expressed as the median (interquartile range of the data). Significant differences in the same row and the same columns (‡, *P* < 0.05; §, *P* < 1e−5; ¶, *P* < 1e−20). Holm’s method was used to adjust the *P-*value for multiple comparisons.

On the other hand, we also wanted to test whether the frequency of PAM sequences was different from what would be expected by chance, taking into account the nucleotide frequency in each phage and plasmid. As a result, it was initially seen that both expected and observed frequencies were below the frequency of PAMs in the *P. aeruginosa* genome (Fig. S4). Furthermore, in the case of subtype I-F, the observed frequency of PAM was lower than expected in both plasmids and phages, with a greater significant difference in the phages recognized by the CRISPR-Cas system. This would support the idea of PAM depletion in genomes of phages recognized by subtype I-F.

### The sequences targeted by subtype I-F contain degenerate protospacers

**Fig. 5.**
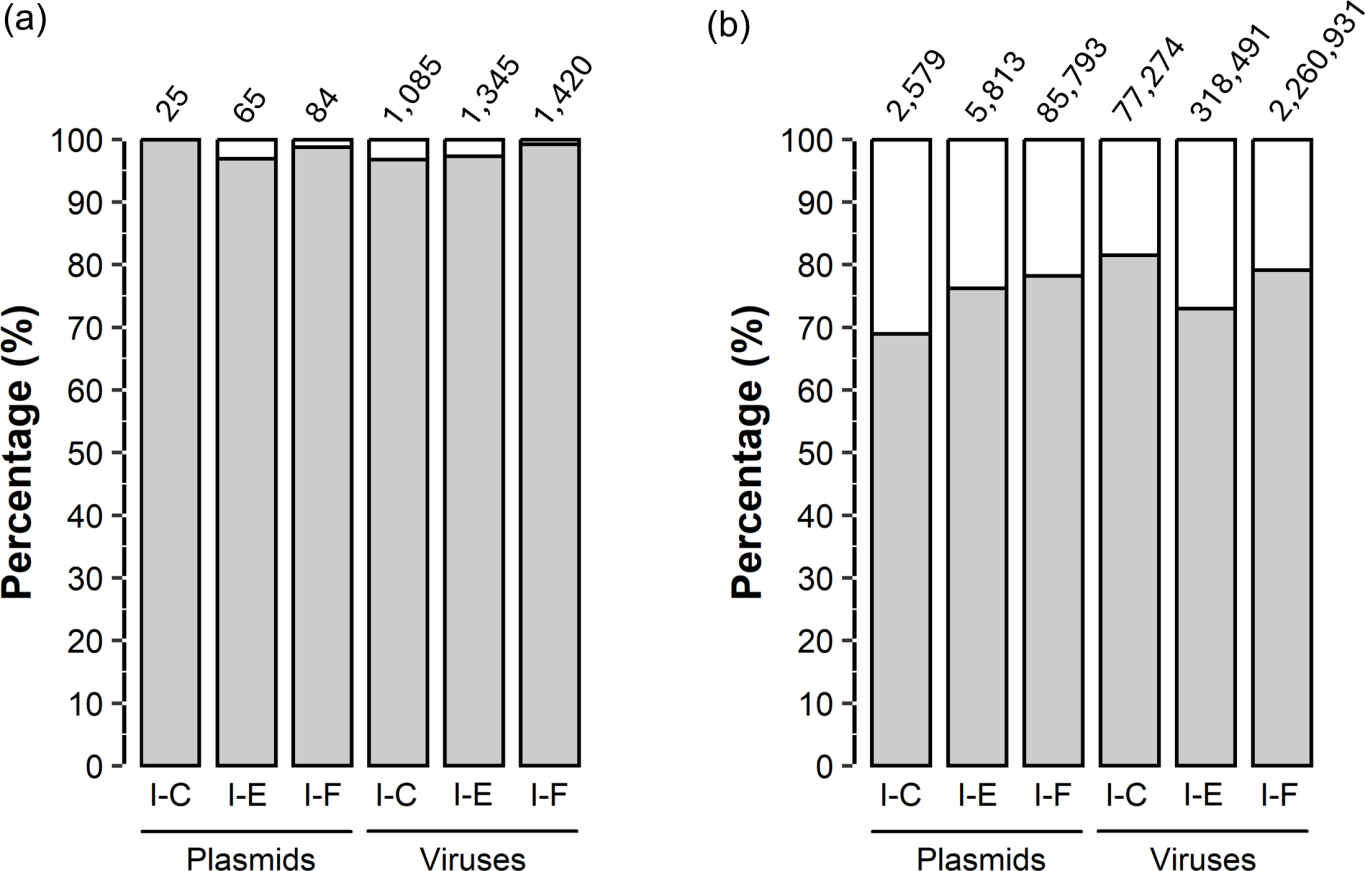
Difference between 95 and 100% identity thresholds for locating protospacers. (**a**) Proportion of viral and plasmid sequences found at 100% identity (grey colour) compared to those found exclusively at identity ≥95% (white colour). (**b**) Proportion of protospacers found at 100% identity (grey colour) compared to those found exclusively at identity ≥95% (white colour). Absolute total values are shown above the bars.

To evaluate possible evasion of the CRISPR-Cas system by the phages via modification of the protospacers, the protospacer search was again performed, but restricting the identity to 100% concerning the corresponding spacer. Thus, the protospacers not found following this new strategy, but previously found at ≥95% identity, could be considered as protospacers that have recently accumulated mutations in their sequence, which could impair their recognition by the spacers acquired earlier in the analysed CRISPR arrays. In this new search at 100% identity, the number of protospacers found decreased to 69–82%; however, the number of phages still retaining at least one protospacer was almost completely maintained (Fig. 5). The results were most striking in the case of subtype I-F, in which only 11 of 1,420 initial phages (0.8%) would now lack 472,024 protospacers (20.9%) at 100% identity, which were found at ≥95% identity, and similar for the plasmids (21.8% protospacers missed in 1.2% plasmids not recognized at 100% identity). Moreover, the PAM 5′-CC occurred at the frequency of 84.8% in the targeted viruses at 100% identity (84.4% at ≥95%). This indicates that mutation of protospacers identified by subtype I-F could occur much more frequently than mutation of the PAM sequence. These percentages of missing protospacers suggest sequence divergence of the protospacers to avoid their recognition by the spacers present in current CRISPR arrays. Furthermore, no significant variations were found in the proportion of mutated PAM sequences between ≥95 and 100% identities (Table S3, Fig. S3).

### The first two positions of the spacers of subtype I-F have a significant underrepresentation of their PAM sequence

**Fig. 6.**
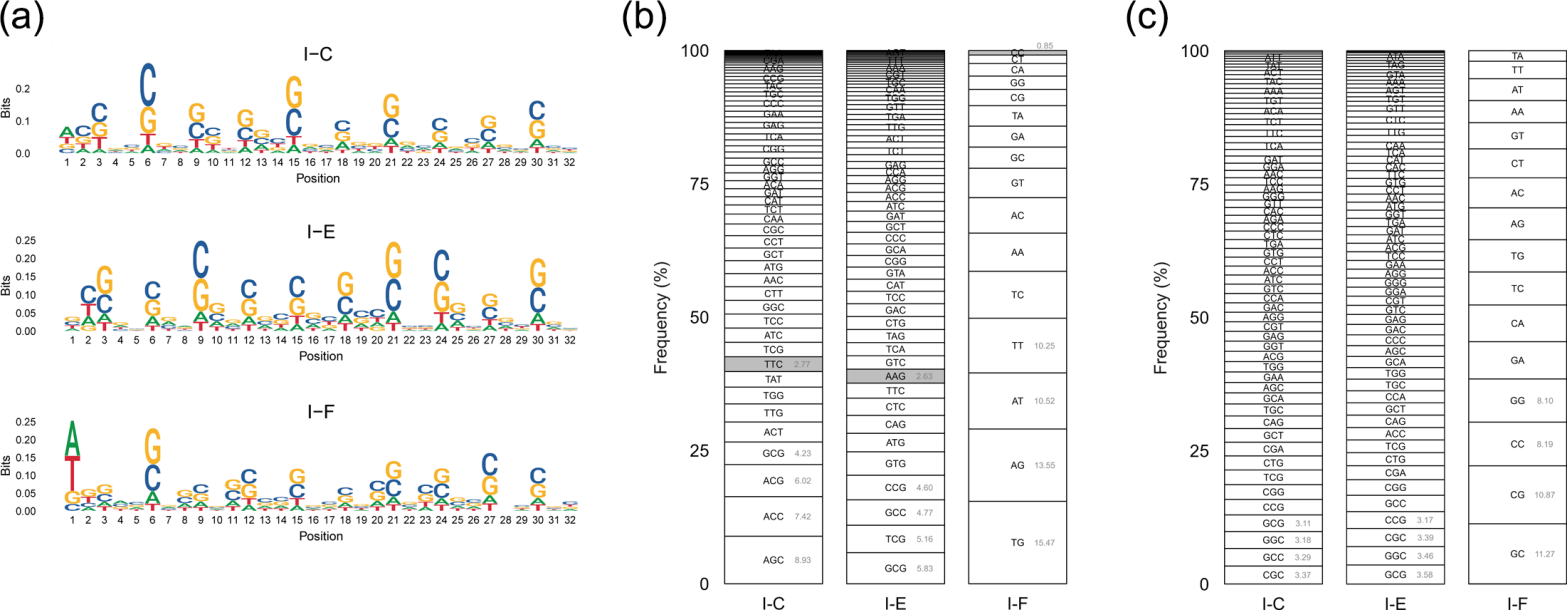
Nucleotide frequency found in the spacer sequence. (**a**) Logo representing nucleotide variability at each spacer position considering a maximum length of 32 nt. (**b**) Frequency of 2–3 nt combinations at the first spacer positions, ordered from most to least frequent. The combination with a sequence identical to the PAM of each subtype is shaded in grey. The four most frequent combinations are accompanied by their relative percentage value. (**c**) Frequency of 2–3 nt combinations throughout the entire spacer sequence, excluding the first two (subtype I-F) or three (subtypes I-C and I-E) positions of the spacer.

Hundreds of thousands of spacers from different CRISPR-Cas systems of *P. aeruginosa* comprised this study, allowing the analysis of their sequences for the identification of conserved patterns. The occurrence of nucleotides along the spacer sequence revealed that cytosine and guanine nucleotides prevailed in many of the positions (Fig. 6a), something that may be due to the high G+C content of *P. aeruginosa* genomes (~66 mol%). However, the position +1 of the spacer had a similar frequency for all four possible residues, except for subtype I-F, where adenine and thiamine were predominant. Considering this result, the frequency of the different combinations of nucleotides of length equal to the PAM predicted for each subtype at the start of the spacer was calculated (i.e. trinucleotides for subtypes I-C and I-E and dinucleotides for subtype I-F). In the case of subtype I-F, the highest frequency observed corresponded to the TG (15.47%), AG (13.55%) and AT (10.52%) dinucleotides (Fig. 6b). However, the combination corresponding to its PAM sequence (5′-CC) hardly appeared (0.85%, chi_square *P*<2.2e−16). This suggests that next to the 5′-CC PAM of this subtype, the reappearance of this same dinucleotide seems to be avoided. On the other hand, the frequency of dinucleotides throughout the rest of the spacer sequence (+3 onwards) presented 5′-TA as the least abundant combination, while the combinations formed by only cytosine and guanine (CC, GG, CG, GC) were the four most abundant, representing 38% of all of them (Fig. 6c).

### The G+C content was lower in the sequences targeted by the I-F subtype

Sequences targeted by a CRISPR-Cas system could enhance their escape through mutations in the PAM sequence, altering the G+C content. In particular, modification of the PAM sequence in the sequences targeted by the I-F subtype, which is composed only of cytosine or guanine (5′-CC or 3′-GG), to adenine or thiamine when subjected to selection pressure would result in a reduced G+C content. This fact was found when comparing the sequences of phages targeted by this subtype, with a G+C content of 60.92 mol% on average, vs. those non-targeted, whose mean G+C content of 62.50 mol% was closer to the 66.5 mol% G+C content of the reference strain PAO1 of *P. aeruginosa* (Fig. 4d). Such reduction of the G+C content in the targeted sequences was significant for both the plasmids (*P*=9.7e−3) and viruses (*P*=1e−7) recognized by the CRISPR-Cas subtype I-F but also for the viruses targeted by the I-E (*P*=9.2e-6), whose PAM sequence included one guanine/cytosine (5′-AAG and 5′-TTC). This suggests that the drop in the G+C content of viruses targeted by the CRISPR-Cas systems could be due to something more than just the loss of consensus PAM sequences 5′-CC. However, it is striking that the protospacers exhibited GC-rich nucleotide combinations in both the trinucleotides of the I-C and I-E subtypes and the dinucleotides of the I-F subtypes (Fig. 6c).

## Discussion

The results of this work have revealed the existence of five subtypes of CRISPR-Cas systems in *P. aeruginosa* that include a high proportion of spacers in their CRISPR arrays. The CRISPR-Cas subtypes described, and their abundance pattern, were consistent with previous studies [24,36]. Approximately 50% of the *P. aeruginosa* genomes were predicted to contain a CRISPR-Cas system of subtypes I-C, I-E, I-F and/or IV [24]. Of these, the CRISPR-Cas subtype I-F remains the most prevalent in *P. aeruginosa* [23]. In contrast, the underrepresentation of the type IV CRISPR-Cas led to its removal from further analysis, its frequency being similar to that reported by Botelho *et al*. [36], who found an occurrence of this system in this host of 5 genomes within a reduced dataset of 1,991 *P*. *aeruginosa* publicly available genomes. In addition, most arrays (*n*=9,557; 97%) had fewer than 30 spacers, which would be consistent with a 2021 review on the dynamics of CRISPR arrays [37]. A higher proportion of spacers per array was found in the *P. aeruginosa* CRISPR-Cas subtype I-C with ∼24 spacers/array, followed by ∼16 and ∼13 spacers/array in CRISPR-Cas subtypes I-F and I-E, respectively. This suggests that CRISPR-Cas subtype I-C may provide an immune advantage to the host due to a higher probability of exogenous DNA destruction without a significant increase in fitness cost [38]. Nonetheless, only 45% of the *P. aeruginosa* genomes had complete CRISPR-Cas systems, while an additional 10% had CRISPR arrays without their cognate *cas* genes, which retain the sequence of the repeats and contain spacers that recognize phage genomes. All this supports the idea of continuous transfer and loss of these systems, probably associated with their need in certain contexts [19].

The present study showed that the *in silico* PAM prediction depends on the availability of CRISPR-Cas arrays and matching plasmid or phage sequences in genomic databases, with increasing PAM prediction scores as the number of CRISPR-Cas systems increases, which enhances the likelihood of finding a target protospacer sequence and, thus, a consensus PAM. In this study, the PAM sequences found were consistent with previous analyses reporting the PAM sequence targeted by *P. aeruginosa* CRISPR-Cas subtypes I-C, I-E and I-F [20,25, 26]. Moreover, the consensus DNA sequence representing the PAM sequence in the plasmids and viruses being targeted by the *P. aeruginosa* CRISPR-Cas subtypes I-C, I-E and I-F coincided with those predicted by Spacer2PAM. This demonstrates that the predicted PAM sequences were valid for the most common CRISPR-Cas systems in *P. aeruginosa* and that the spacers constituting the CRISPR arrays originated from plasmids and viruses that previously infected these bacterial strains. Furthermore, the information content at the known positions reveals that despite CRISPR-Cas subtype I being specialized in phages, this subtype could also be able to recognize plasmids, suggesting the possible role of these sequences as phage–plasmids.

The abundance of diverse PAMs with distinct nucleotides at the known PAM positions seemed to be more equitable in plasmids than in viruses. Besides, a higher diversity of PAMs was found in plasmids than in viruses, in proportion to the number of different targets found. This means that plasmids and viruses could escape immunity by acquiring mutations in the PAM, although according to the findings here to a greater extent in plasmids, which could compromise the effectiveness of the immune system of the bacterial host during recognition of foreign sequences as they would need to acquire a new spacer from a plasmid or virus with a mutated PAM to regain immunity. Furthermore, the subtype of the CRISPR-Cas system that *P. aeruginosa* carries might provide different immune advantages against exogenous genetic material. Indeed, the protection by the CRISPR-Cas system is limited by the diversity of targets being recognized by these adaptive immune systems. This study showed a higher heterogeneity of targets being recognized by *P. aeruginosa* CRISPR-Cas subtype I-F, followed by CRISPR-Cas subtype I-E and subtype I-C, although CRISPR-Cas system I-E showed more similarity to CRISPR-Cas system I-F than to CRISPR-Cas system I-C. This might be the result of decreasing abundances of each of these systems, with plasmids and viruses able to infect *P. aeruginosa* strains harbouring CRISPR-Cas systems of different subtypes. Therefore, the recognition of plasmids or viruses is not limited to specific CRISPR-Cas systems, but each of these adaptive immune systems offers limited protection. As previously suggested, this may indicate a negative correlation between plasmid and viral diversity and CRISPR-Cas efficacy [39]. At the same time, molecular memory of past infection can be stored more than once in the same CRISPR array as different spacers match the same plasmid or virus. Furthermore, it must be considered that the frequent recombination between phage genomes can occasionally lead to phages not initially recognized by a CRISPR-Cas system being targeted when they acquire the protospacer sequence from a second phage.

The distribution of the consensus PAM sequences in the target sequences revealed a significantly lower occurrence in the viruses being targeted by the I-F CRISPR-Cas systems compared to the non-targeted viruses, and a significant reduction in their G+C content, which could be a consequence of the accumulation of mutations to escape from the host’s immune system [29]. Moreover, the G+C content of the phages that infect *P. aeruginosa* was predicted to be lower than that of the host genome (65–67 mol%), which agrees with several studies and indicates notable dissimilarities in the codon usage between exogenous viruses and the host *P. aeruginosa* [40,42]. Furthermore, the infecting exogenous sequences might mutate their PAM to escape from being recognized by the host CRISPR-Cas system, especially for those being identified by the *P. aeruginosa* CRISPR-Cas subtype I-F, as the position −1 in the PAM sequence is prone to change to thymine or adenine, reducing the G+C content. However, the results indicated that the PAMs were frequently located upstream of the protospacer. For these reasons, the foreign sequences with a higher occurrence of the PAM sequences might have a higher probability of being recognized by the CRISPR-Cas systems, although the location of the PAM sequences at a specific position would be enough and essential for efficient foreign DNA recognition. All this suggests that the decrease in the G+C content found in this study would also decrease the probability of occurrence of new PAM sequences for the I-F system, even though the spacers (and thus the protospacers from the target sequence) are rich in G+C content, with dinucleotides composed exclusively of guanine and cytosine being the most abundant in their sequence. However, this abundance of guanines and cytosines was opposite in the first two positions of the spacers of this subtype I-F, as the CC dinucleotide seemed to be avoided right next to the PAM sequence. This suggests that this dinucleotide could be excluded at the beginning of the spacer to prevent the possible sliding of the interference complex and hence the recognition of an erroneous PAM next to the correct one. The first position of the spacer has a high ratio of adenine to thymine, as has been previously proven for the I-F subtype [43]. In addition, the guanine or cytosine at position −1 in the PAM sequence recognized by subtype I-E (5′-AAG and 3′-CTT) could point to changes at the closest nucleotide to the protospacer, which could also support the escape of this CRISPR-Cas system. This idea agrees with what has already been observed with the *Escherichia coli* CRISPR-Cas subtype I-E using the CRISPR-Cas system both endogenously in bacteria and to transform human cells [44,45]. Specifically, the PAM variants that still give detectable activity retain C or G at position −1 (5′-ATG, 5′-AGG, 5′-GAG, 5′-TAG and 5′-TAC).

The difference found between the subtypes of CRISPR-Cas type I system could be due to the selection pressure exerted by subtype I-F in this species, with 126,057 spacers distributed in 3,928 genomes, while the other two subtypes only comprised up to five times less (25,762 distributed in 1,016 genomes). This selective pressure that would exert such a large number of genomes on the target phages could explain why only the escape of phages from the subtype I-F can be predicted by divergence of their PAM sequence, since the strategy of making the CRISPR-Cas system more abundant in the bacterial population would help to eliminate the phage from the environment [46]. Something to note here is that mutations in the PAM sequence of phages and plasmids would not only prevent the interference step of the CRISPR-Cas system and thus the elimination of the foreign sequence, but also the proper functioning of the adaptation step, thus preventing the creation of new spacers against the target sequence. However, this could be countered by the so-called CRISPR adaptation priming, whereby previous CRISPR array spacers can help to add new spacers, even if they have mutations in the protospacer sequence or the PAM sequence [47]. Thus, at least in the case of the frequent CRISPR-Cas subtype I-F of *P. aeruginosa*, it can be confirmed that phages that are targeted by this defence system would benefit from mutations in their PAM sequences, which would secondarily reduce their G+C content. It should be noted that bias in G+C content is common in mobile genetic elements of bacteria, and this may be due to multiple causes [48]. However, PAM depletion has been described as a selective advantage for phages to escape from CRISPR-Cas systems in subtypes I-C and I-E, although using a low number of genomes in phages infecting the genera *Streptococcus* and *Vibrio* [49]. Furthermore, it has also been well-established in other defence systems, such as restriction-methylation systems, in which mutations that prevent palindromicity of target sequences also allow phage escape [50].

## Conclusion

In conclusion, the CRISPR-Cas subtype I-F remains the predominant *P. aeruginosa* CRISPR-Cas system in this host, which is characterized by its ability to recognize the highest number of foreign invaders, while CRISPR-Cas subtypes IV-A1/2 are rarely found in the bacteria. This study evidenced that the spacers within *P. aeruginosa* CRISPR arrays originated from foreign sequences that previously infected the bacterial host. Among the existing CRISPR-Cas systems in *P. aeruginosa*, type I might be oriented to viruses, although it can also target plasmids. Furthermore, the different occurrence of the consensus PAM sequence in the foreign sequences might influence their recognition by the CRISPR-Cas systems, especially for subtype I-F, where reduced PAM frequencies in the foreign sequences being targeted by the CRISPR-Cas systems were observed in combination with reduced G+C content, which would be the result of selection pressure on the PAM. Nonetheless, the consensus PAM sequences were predominantly located in the region upstream of the protospacer, highlighting that this region contains the PAM, which is sufficient and essential for efficient foreign DNA recognition.

## Supplementary material

10.1099/mgen.0.001423 Supplementary Material 1.

10.1099/mgen.0.001423 Supplementary Material 2.

## Abbreviations

CRISPR-Cas: clustered regularly interspaced short palindromic repeats and CRISPR-associated
crRNAs: CRISPR RNAs
NCBI: National Center for Biotechnology Information
PAM: protospacer adjacent motif
