# Supplementary material for: Bacteriophages in Pseudomonas aeruginosa evade the CRISPR-Cas I-F system by depletion of PAM sequences
Source: Microb Genom. 2025 Jun 17;11(6):001423. doi: 10.1099/mgen.0.001423 (PMC12174588; doi:10.1099/mgen.0.001423)
Supplement: Supplementary Material 2. [file mgen-11-01423-s002.pdf]

Bacteriophages in *Pseudomonas aeruginosa* evade the CRISPR-Cas I-F system by depletion of PAM sequences

Supplementary Figures

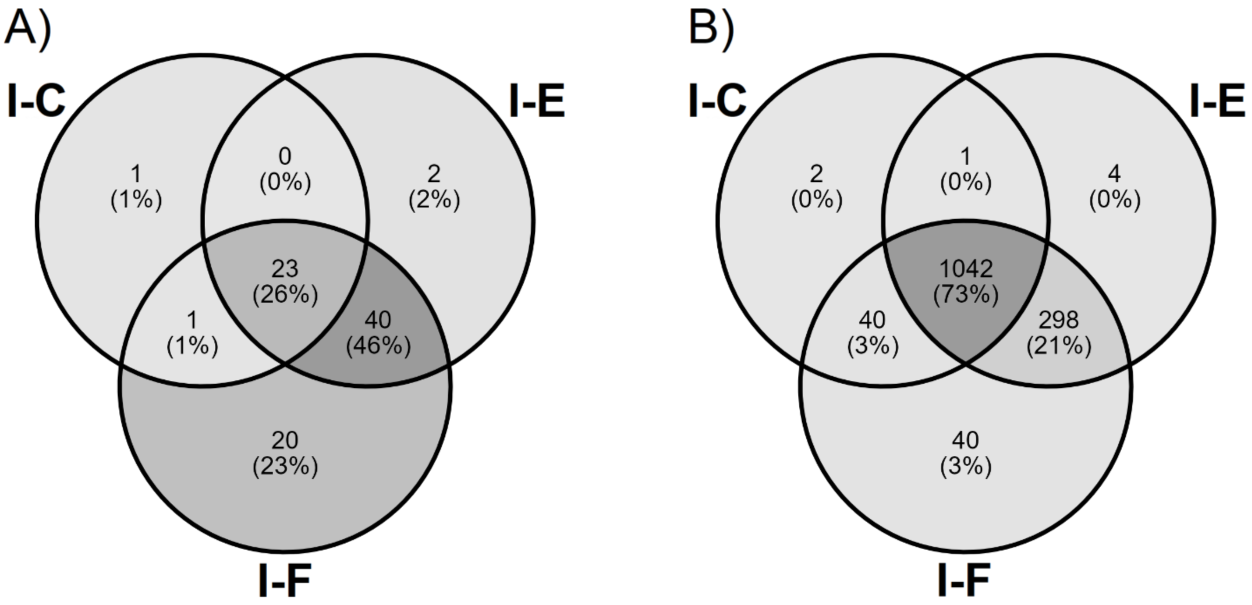

Supplementary Figure S1. Distribution of plasmids (A) and viruses (B) being targeted by the *P. aeruginosa* CRISPR-Cas subtypes I-C, I-E and I-F.

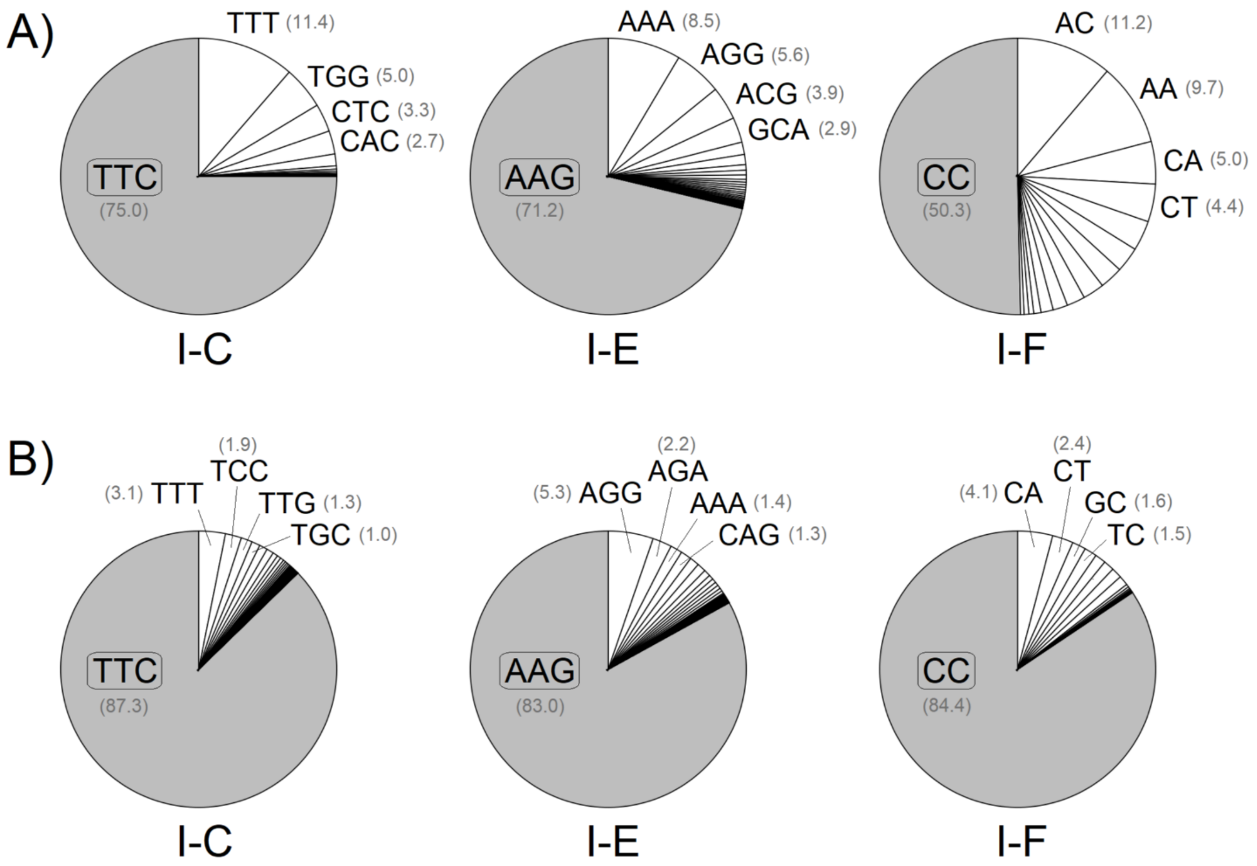

Supplementary Figure S2. Abundance (%) of the different PAM sequences of length matching that predicted by Spacer2PAM that were found in the plasmids (A) and viruses (B) targeted by the *P. aeruginosa* CRISPR-Cas subtypes I-C, I-E and I-F. The most frequent PAM sequence for each CRISPR-Cas system is shown in gray (5'-TTC for subtype I-C, 5'-AAG for subtype I-E, and 5'-CC for subtype I-F), while other PAMs are shown in white. The top-4 more frequent mutated PAMs are labelled. Frequency for all PAM sequences identified can be found in Supplementary Table S3.

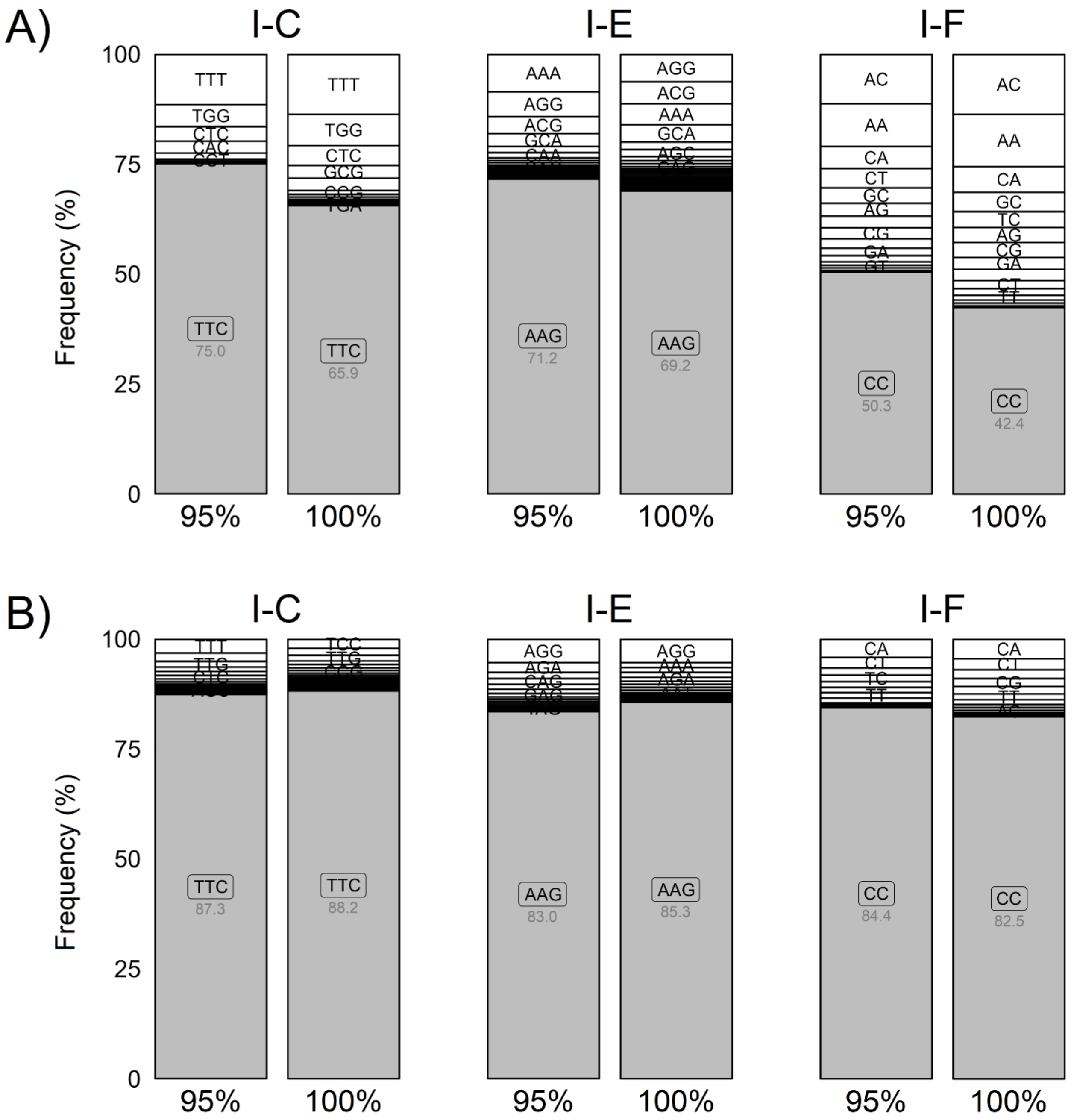

Supplementary Figure S3. Abundance (%) of the different PAM sequences of length matching those predicted by Spacer2PAM found in the (A) plasmids and (B) viruses targeted by the *P. aeruginosa* CRISPR-Cas systems. Targeted plasmids and viruses were defined as those with a sequence identity ≥ 95% (or 100%) and a query coverage = 100% against spacers of a *P. aeruginosa* CRISPR-Cas system, respectively. Frequency for all PAM sequences identified can be found in Supplementary Table S3.

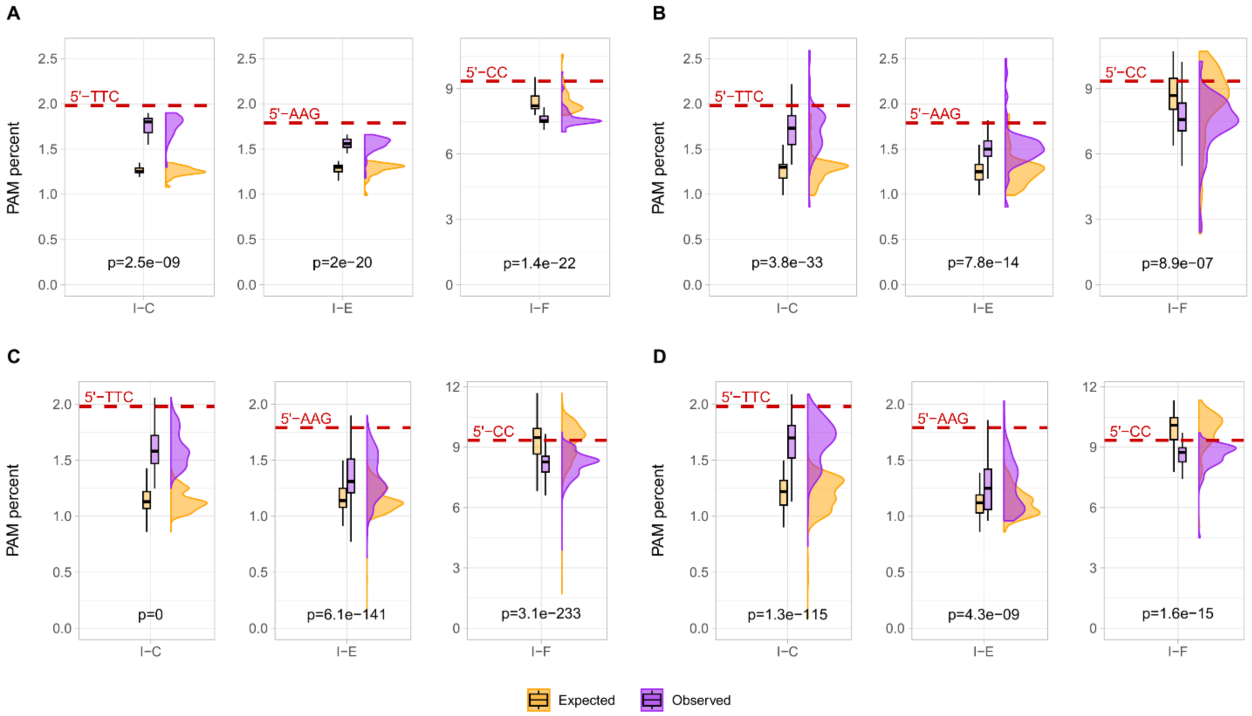

Supplementary Figure S4. Distribution of PAM expected and observed frequencies in the groups of targeted and non-targeted sequences by the *P. aeruginosa* CRISPR-Cas subtypes I-C, I-E and I-F. (A) Targeted plasmids, (B) Non-targeted plasmids, (C) Targeted viruses, and (D) Non-targeted viruses. Dashes lines show the PAM frequency in the *P. aeruginosa* reference strain PAO1 (GCF_000006765.1). The Holm method was used to adjust the p-value for multiple comparisons. The numerical values can be found in Supplementary Table S4.
